# Influence of Dietary Protein Source and Level on Histological Properties of Muscle and Adipose Tissue of Lambs

**DOI:** 10.3390/foods12061284

**Published:** 2023-03-17

**Authors:** Davide De Marzo, Caterina Losacco, Vito Laudadio, Vincenzo Tufarelli, Youling L. Xiong

**Affiliations:** 1 Department of Precision and Regenerative Medicine and Jonian Area, Section of Veterinary Science and Animal Production, University of Bari ‘Aldo Moro’, 70010 Valenzano, Italy; 2 Department of Animal and Food Sciences, University of Kentucky, Lexington, KY 40546, USA

**Keywords:** lamb, diet, muscle fiber types, adipose tissue, cell size

## Abstract

The muscle and adipose tissue histological properties in wether and ewe lambs of Gentile di Puglia breed, fed diets including two protein sources [soybean meal (SB) and SB plus distillers dried grain with solubles (DD)] and three protein levels (12.5, 15.7, and 18.9%) were evaluated. Muscle samples were collected from the longissimus/rump, cut, and stained (reciprocal aerobic and anaerobic stains) for muscle fiber typing and fat cell characterization. Fibers were classified as α-red, β-red, and α-white. Lambs fed SB had larger α-white (*p* < 0.10) and smaller-diameter β-red and α-red fibers (*p* < 0.05). Among dietary protein levels, lambs fed 12.5% protein exhibited the highest percentage of α-red and the greatest diameter of α-white fibers, whereas wethers had a higher percentage of α-red (*p* < 0.05), and ewes had a higher percentage of α-white fibers (*p* < 0.05). Intramuscular fat cells were larger (*p* < 0.10) in ewes than in wethers. Lambs in the group fed 12.5% protein had larger subcutaneous fat cells at the sacral vertebrae location. Overall, both sources and levels of dietary protein had significant effects on lamb muscle and fat histological features, suggesting the potential of modulating muscle or fiber types through dietary protein strategies.

## 1. Introduction

Lamb is an important source of meat that is widely consumed worldwide, and it is considered essential in many countries for cultural and ethnic reasons. According to the OECD-FAO [1], approximately 15 M tons of lamb meat was consumed, and the level is expected to increase to 16 M tons by the end of 2023. Small ruminants are the prevalent species of domestic livestock animals in the Mediterranean region. In many areas of the region, sheep are commonly reared under semi extensive or extensive conditions according to pasture characteristics. In these areas, an ovine rearing system is conducted traditionally by using local sheep breeds due to their exceptional adaptation to environmental conditions and utilization of existing feed resources.

The number and size of muscle cells present in the body of animals are important for meat production. It has been recognized that diversity of muscle fibers (i.e., number, diameter, and type) have a significant effect the qualitative traits of meat [2]. Metabolic and contractile characteristics are important manifestations and predictive factors for the heterogeneity of muscle fibers. In fact, skeletal muscle fibers are normally classified based on selective cellular components that are directly involved in the specific contractile and metabolic activities. In the sheep fetus, muscle fiber numbers are complete at approximately 80 days of gestation [2,3]. These numbers are determined genetically and have heritability estimates of 0.17–0.38.

In fresh muscle immediately post-mortem, adenosine triphosphate (commonly referred to as ATP) is produced through the anaerobic glycolytic pathway from glucose and stored glycogen, and this is a part of the post-exsanguination biochemical conversion of muscle to meat. By a natural process, lactic acid, the final product of glycolysis, is accumulated in muscle due to the cessation of blood circulation [2]. If the glycolytic fibers are dominantly distributed in an individual muscle, rapid post-mortem glycolysis occurs. Conversely, if a muscle is predominantly comprised of oxidative fibers, less lactic acid is produced due to the deficiency of substrates (glucose and glycogen). The accumulation of lactic acid results in a rapid muscle pH decrease reaching an ultimate level of 5.4–5.8, depending on the relative preponderance of different fiber types in the muscle tissue. Therefore, the composition of muscle fibers is rather crucial in regards to the post-mortem metabolism pattern and glycolytic products during muscle to meat conversion, which ultimately affects the qualitative traits of meat.

The importance of muscle fiber types in studying meat characteristics is mainly related to two aspects: firstly, muscle fiber growth and development reflect the general growth curve pattern of livestock animals and therefore the ultimate body size; secondly, the final product (meat) to be served to consumers is of great interest since it determines meat acceptability based on organoleptic evaluations [4]. It was reported that a significant correlation between βR fiber size and carcass juiciness and tenderness scores existed in lambs. Moreover, it is well established that muscles with different physiological functions generally differ in metabolism. Being tonically active, red-type skeletal muscle generally exhibits a higher rate of oxidative metabolism than white-type skeletal muscle. However, most skeletal muscles are a mixture of red and white fibers; the relative percentages vary with anatomic locations on the carcass as well as with rearing conditions, genetics, feeding management, and amino acids composition of feeds.

A deeper understanding of production factors that influence the physiology of muscle growth and development is important not only from the perspective of carcass yield but also in terms of meat quality and sensory properties. Many authors have reported that the size of fibers in sheep increased with age, sex, exercise, and improved nutrition [5]. Moreover, different sources of energy in lamb rations appeared to cause a physiological shift from intermediate muscle fibers to white muscle fibers [6]. These authors suggested that the ATPase activity may not be fixed at birth.

In skeletal muscles, there are two basic fiber types: alpha (α) and beta (β) [7]. The beta fibers are “red” (β-red) and generally do not change, whereas alpha fibers are initially “red” but may be transformed from an alpha red (α-red) to an alpha white (α-white). Ashmore et al. [7] suggested that the increase in muscle size is due in part to the conversion of smaller α-red fibers to larger α-white fibers. Johnston et al. [8] reported that the percentages of intermediate and white muscle fibers decreased as the protein content in the ration increased, and Facciolongo et al. [9] indicated that the protein sources did not influence the physical characteristics of the meat. Furthermore, it appears that various types of dietary restrictions may have a selective effect on one muscle type but not another.

An increased fat deposition is due both to hyperplasia and hypertrophy of adipocytes [9,10,11,12]. In sheep, subcutaneous fat depots increased in response to hyperplasic growth between 8 and 14 months of age [13]. Numerous researchers have reported that adipocytes in porcine subcutaneous fat tissue do not develop uniformly; instead, they accumulate lipid at different stages of growth [14,15,16,17]. For example, in bovine muscle, intramuscular fat cells have been found to differentiate as clusters of varying sizes [18].

Despite the general knowledge of the role of dietary proteins in muscle cell development and growth, few studies had specifically compared different source and level proteins on muscle fiber characteristics in lambs. Therefore, the present study was conducted to evaluate the effects of different dietary protein sources and levels on the histological properties of lamb muscle and subcutaneous fat.

## 2. Materials and Methods

### 2.1. Lamb Production and Harvest

Thirty-six lambs of Gentile di Puglia breed were weaned (40 ± 2.0 days old) at approximately 13.5 ± 0.45 kg of body weight and allocated in equal numbers to six dry lot feeding regimes (Table 1). Lamb groups 1, 2, and 3 received a corn-soybean meal (SB) diet, which contained 12.5, 15.5, or 18.9% crude protein, respectively, until the slaughter weight of approximately 20 kg (at 70 days of age). Groups 4, 5, and 6 received the same three levels of protein, but with distillers dried grain soluble (DD) replacing part of the SB.

The total mixed rations (TMR) as pellets were formulated to meet the nutrient requirements for lambs and to be isocaloric according to Laudadio and Tufarelli [19]. Diets were formulated to contain a mean of 9.21 MJ/kg of dry matter (DM) of metabolizable energy (ME) utilizing feed analysis in each treatment. For forages, the rations (oat hay) were re-chopped by grinding at 25 mm and subsequently mixed and pelleted by the team (ca., 8 mm in diameter) to maintain the integrity of fibrous elements. This process was done to reduce differences in physical form and prevent the feed selection bias of the experimental lambs. The TMR were provided to animals in two equal meals. Feed rationing was applied to avoid unnecessary feed ingestion by subjects, which in previous trials had led to issues of lambs’ death. Clean drinking water was available ad libitum. The body weight (BW) of each lamb was recorded weekly prior to feeding at 07:00 h. The feed conversion ratio (FCR) was assessed as the ratio of BW gain to DM intake. Refusals of feed were sampled daily, weighed, and individually analyzed. The samples of collected refusals along with the feed offered to each animal were dried at 105 °C for 24 h to determine DM intake. Before the study was started, all lambs were inoculated against clostridial infections and treated for internal parasites. During the course of the feeding trial, lambs were regularly observed for health and well-being by a veterinarian. At the end of the feeding trial, lambs were humanely harvested according to the University Institutional Review Board protocol. Carcasses were chilled in a 3 °C walk-in cooler for approximately 48 h and then evaluated and sampled.

### 2.2. Muscle Sample

Lamb carcasses were evaluated and then fabricated into wholesale cuts. The measurements included the depth of fat over the spinous process, the depth of fat over the tail (rump), and the depth of fat over the leg (30 cm from the shank end). Samples for histological examination were obtained from the center of the longissimus (left side at the 13th rib) and the base of the tail (rump) over the sacral vertebrae. Duplicate 1 cm^3^ samples were immediately immersed in liquid nitrogen.

Frozen muscle samples were mounted on a Cryostat chuck so that fibers were perpendicularly oriented to the blade. After a 20 min equilibration at −20 °C, the samples were sectioned to 16 µm thickness using a Damon freezing microtome-cryostat (Damon/IEC Division, Damon Corp., Needham Heights, MA, USA). Serial sections were mounted on glass microscope slides, allowed to air dry, and then stained with NADH-TR. Samples were myofibrillar ATPase reacted and treated with Oil-Red-O and Hematoxylin [20] at alkaline pH [21]. Once the tissue section was stained, a microscope cover slip was placed over the tissue section and fixed in place with glycerol jelly.

The sample slides were observed under a Zeiss photomicroscope (Carl Zeiss, Inc., New York, NY, USA). Several fields of each stained section were photographed at 25× magnification with the bright field setting on the light microscope. A stage micrometer with 0.01 mm graduations was also photographed for size definition and scaling. The photomicrographs were enlarged to a 12.7 × 17.8 cm dimension to facilitate the analysis and differentiation of fat cells and muscle cell types.

Muscle cells were typed on the basis of staining reaction into red (α-red), intermediate (β-red), and white (α-white) types. All fibers inside a field size (6 × 4 cm) were counted and then measured using a Zeiss particle size analyzer (Carl Zeiss, Oberkochen, Germany). An enlarged photograph of the micrometer scale was also measured. This micrograph was used in the conversion of the instrument values to maximum round diameter (MRD) of the cell (µm) [5]. The MRD for each fiber type was calculated based on the following equation: fiber diameter (micrometers) = actual value for micrometer scale/instrument value for micrometer scale X instrument value for fiber × 1000.

In addition to fiber diameter, the percentage of cells for each of the three fiber types was calculated by dividing the number of each type by the total number of counted cells. For the measurement of fat cells, the photomicrographs of Oil-Red-O slides were used. The MDR of fat cells were measured on each sample, and the values were converted to µm by the same procedure used for muscle fibers as described above.

### 2.3. Statistical Analysis

Data were analyzed by the least-squares procedure assuming a mathematical model that included the fixed effect of protein source, protein level, and the protein source × protein level interaction. The pen within protein source × protein level was included as a random effect. Differences among means were tested for significance using the protected least significant difference procedure [22].

## 3. Results and Discussion

Meat consists of countless tissues, predominantly muscular tissues, which are composed of muscular fibers. These fibers are the basic constituents of skeletal muscle and could be divided into different types. The relative distribution and changes in the types of muscle fibers during sheep production have a direct impact on the quality of lamb meat. For example, red fibers are of a higher myoglobin content (color), generally higher pH, and more taste-impactful nucleotides than white fibers [4]. To the best of our knowledge, very limited literature has been published in recent years on the effects of different dietary protein sources and levels on the histological properties of lamb muscle and subcutaneous fat. The consumer demand for ovine meat is on a steady rise, which underscores the need for new and further investigations.

Soybean (SB) meal is an important protein source for animal nutrition; however, the use of this conventional ingredient increases feed costs [23]. Thus, using agricultural by-products, such as DD, as a protein source to replace SB can reduce costs as well as ruminal protein degradation [24]. However, it seems that the feed value of DD may vary according to the inclusion level [25]. Generally, DD have mostly been fed to beef and dairy cattle, swine, and poultry [26]. Even though DD should be appropriate for lambs, the feeding value of DD in finishing diets fed to lambs is not well-defined because only a limited amount of research evaluated the use of DD in lamb rations [27]. However, given the physicochemical properties of DD, it is being used in finishing diets for feedlot lambs to partially replace corn and SB.

Accordingly, the present study was conducted to test the efficacy of dietary DD at different protein levels in comparison with SB for the modification of muscle fiber types in lambs. The least-square means for size and population of muscle fibers in the longissimus muscle of lambs according to dietary protein source and level are presented in Table 2. For comparing the levels of protein, larger fibers were noted in lambs fed lower percentages of protein, but the differences were significant (*p* < 0.05) only for the white fibers. This was in agreement with Wang et al. [28] who reported that the diameter of muscle fibers in the medium protein group (12.1%) was significantly larger than that in the low protein group (10.1%), whereas the density of muscle fibers showed the opposite trend.

The protein source obviously affected α-white fibers with fibers of the SB lambs being larger (*p* < 0.10). Although no significant difference (*p* < 0.05) was found between sexes, the mean values for α-red (36.6 µm) and α-white (42.3 µm) were the largest for wethers, whereas the mean value for β-red fiber (43.4 µm) was higher for ewes. One interesting observation was that the β-red and α-white fibers were similar in diameter, but both types were larger than α-red fibers. These results, however, are not in agreement with several other studies reported in the literature. Gauthier [29] reported that βR fibers had the smallest and α-white the largest diameter. Moody et al. [30] claimed that the βR fibers were larger than the αR and α-white fiber types. In this study, increasing the protein level slightly decreased the fiber diameters. The percentage of α-red fibers decreased, and the percentage of α-white fibers increased in the longissimus as the protein content in the ration increased (*p* < 0.05) with no significant (*p* > 0.05) difference for β-red fibers.

Johnston et al. [31] reported that, in general, as the energy level in the ration increased, the percentage of intermediate muscle fibers decreased and the percentage of α-white muscle fibers increased. Ashmore et al. [7] concluded that α-red fibers have the capacity to transform into α-white fibers, and Meunier et al. [32] showed that muscle fibers were dynamic structures that can switch from one type to another. Therefore, from the results of this study and those presented by other researchers, it would seem that increasing the protein level influences the transformation from α-red to α-white fibers. In the present study, the effect of protein source on the number of muscle cells (fibers) was limited to β-red fibers (*p* < 0.05) as the longissimus of the DD lambs contained 8.92% βR fibers compared to 7.39% for the SB lambs. Sex influenced the percentage of the α-red and α-white fibers (*p* < 0.05) with wethers having 58.0% α-red fibers compared to 52.0% for ewes, whereas ewes had a higher percentage (40.5% vs. 33.2%) of α-white fibers (*p* < 0.05). These data support the premise that fiber differentiation, that is, conversion from α-red to α-white, accompanies physiological maturity.

The influence of diet on lamb muscle fibers extends to other nutrients than proteins. As reported by Santello et al. [33], the semitendinosus muscle of ½ Dorper-Santa Inês lambs finished in different feeding systems (confinement and grazing plus oil supplementation) had the largest diameters for oxidative-glycolytic fibers (43.7 μm), followed by glycolytic fibers (36.0 μm) and oxidative fibers (20.3 μm). Lambs fed on sunflower grain (9.10%) presented similar-sized diameters for red fibers (35.4 μm), intermediate fibers (36.0 μm), and white fibers (35.3 μm) in relation to the semitendinosus muscle. However, for the Longissimus lumborum muscle, the diameters of the different fiber types exhibited some differentiation, with values of 28.7, 29.8, and 32.2 μm for red, white, and intermediate fibers, respectively [34]. The results suggested that the effect of diet is rather complex, although the type and amount of protein in the diet is important, other nutritional factors could have a contributing role. Therefore, the inter-relationship warrants further investigations.

The least-square means for fat thickness in longissimus, leg, and rump are presented in Table 3. There were no differences among groups due to protein levels for longissimus or leg fat. However, some differences, albeit not linear, occurred in rump fat. It would be difficult, however, to conclude that such differences were due to protein level when no differences occurred over the longissimus or on the leg. When protein sources were compared, the fat over the leg was greater (*p* < 0.05) for the SB lambs. Since the actual difference was small and no significant differences were observed for the longissimus or rump, it is doubtful if the leg difference is meaningful. Fat thickness was greater (*p* < 0.05) over the longissimus for ewes than for wethers, which agrees with Gutiérrez-Peña et al. [35]. Also, Ahmad et al. [36] reported that female lambs had the highest fat mass and adipocyte.

The least-square means for subcutaneous fat cell number and diameters are given in Table 4. As reported by other researchers, fat cell diameters generally increased with the level of energy in diet [37]. As dietary energy levels increase, the body fat normally increases with a concomitant increase in fat cell size and in number [11]. However, an increase in dietary protein, as used in this study did not appear to have a consistent effect on fat cell diameters. The fat cell diameters were larger (*p* < 0.10) in ewe lambs, which were closer to physiological maturity and also fatter than wether lambs. Total intramuscular fat is due both to the number and size of fat cells. Size could be measured but, because of the uneven distribution of fat cells, it was difficult to quantify fat cell numbers. The lower cell numbers for both longissimus and rump were found in the 12.5% protein group. The number of fat cells in longissimus increased for the 15.7% protein group and decreased for the 12.5% group. However, in the rump, the population of fat cells increased for the 15.7 and 18.9% groups and the changes were (*p* < 0.05). All diameters followed an inverse pattern. Protein source affected neither the number nor size of cells from either location.

Sex affected both size and number of fat cells with ewes having larger cells (*p* < 0.05 for the longissimus) and wethers having more cells (*p* < 0.05) for rump, resulting in partial accord with those of Facciolongo et al. [38]. These authors found that diet × sex interaction had a significant impact on the carcass fat incidence, and this was unchanged by sex in the lambs fed on SB meal. The influence of sex on the intramuscular fat proportion appears to be quite controversial. In previous research [39], there was no difference in the relation to sex for slaughtered lambs at a similar body weight as in the present study. Nevertheless, other authors [40] observed a higher proportion of fat muscle in females, ascribing this to the greater predisposition in females to build up fat at an earlier age, and to their slower growth level, which subsequently means that they reach the slaughter body weight later. Moreover, it was reported that the differences between sexes were more evident in suckling lambs than in fat lambs [41]. Furthermore, Bloor et al. [42] concluded that sex had a major impact on body fat distribution, and males were more susceptible to visceral adiposity and obesity-related diseases than females, although the underlying mechanisms for these gender differences were not well understood. This was probably due to the difference in maturity as the ewes at equal weight were closer to physiological maturity. Further research is required to gain insight into the molecular and cellular mechanism(s).

## 4. Conclusions

Based on the results of the present study, it can be concluded that the dietary protein source fed to lambs had significant effects on the characteristic of muscle fibers; in particular, on α-white fibers in both ewe and whether with respect to fiber size and distribution. Moreover, the lamb sex significantly influenced the features of subcutaneous fat tissue. Further, both sources and levels of dietary protein had a measurable effect on lamb muscle and fat histological characteristics. Of special note is the apparent conversion of α-red (intermediate) fibers to α-white fibers in lambs, especially in ewes, corresponding to increasing amounts of protein feed from distillers dried grain with soluble meal. These findings are significant for lamb nutrition and meat quality when alternative sources of proteins (soybean meal) are considered in lamb production. More research is warranted to deeply verify the present findings as well as to identify other nutrition factors, such as feed amino acids profile, that may contribute to fiber type differentiation. Overall, understanding the dietary protein influence on lamb muscle fiber types, fat deposition and characteristics, and gender difference, as demonstrated in the present study, may be valuable for the design and implementation of production strategies to optimize the quality of lamb meat.

## Figures and Tables

**Table 1:** Experimental design.

	Protein Source			
	Soybean Meal (SB)		Distillers Dried Grain with Solubles (DD)	
Protein level, %	Wether	Ewe	Wether	Ewe
12.5	3	3	3	3
15.7	3	3	3	3
18.9	3	3	3	3

**Table 2:** Least squares means and standard errors for size and population of muscle fibers and size of fat cells in longissimus muscle ^a^.

Item		Muscle Fiber Diameter, µm			Populations, % of Total		
	No. Lambs	β-Red	α-Red	α-White	β-Red	α-Red	α-White
Protein, %							
12.5	12	43.0 ± 2.0	36.8 ± 0.9	43.8 ^d^ ± 1.9	8.8 ± 0.1	60.0 ^d^ ± 2.9	31.2 ^e^ ± 2.8
15.7	12	42.2 ± 2.0	36.6 ± 0.9	42.5 ^e^ ± 1.9	7.9 ± 0.8	56.4 ^e^ ± 3.0	35.7 ^d^ ± 2.8
18.9	12	42.7 ± 2.1	35.6 ± 1.0	41.4 ^e^ ± 2.1	8.1 ± 0.8	55.2 ^e^ ± 3.0	36.8 ^d^ ± 3.0
Source							
SB ^b^	18	43.6 ± 1.4	35.4 ± 0.6	42.8 ^d^ ± 1.4	7.4 ^e^± 0.6	57.1 ± 2.0	35.5 ^e^ ± 2.0
DD ^c^	18	41.5 ± 1.5	35.5 ± 0.7	40.9 ^e^ ± 1.4	8.9 ^d^± 0.6	52.9 ± 2.1	38.2 ^d^ ± 2.1
Sex							
Wether	18	41.6 ± 1.5	36.6 ± 0.7	42.3 ± 0.6	8.8 ± 0.6	58.0 ^d^ ± 2.1	33.2 ^e^ ± 2.1
Ewe	18	43.4 ± 1.4	36.4 ± 0.6	41.4 ± 0.6	7.5 ± 0.6	52.0 ^e^ ± 2.0	40.5 ^d^ ± 2.0

^a^ Classification is based on reaction with NADH-TR and myofibrillar ATPase alkaline pH; Fiber type number expressed as % of total number of fibers within 24 cm^2^ area; ^b^ SB = Soybean meal; ^c^ DD = Distillers dried grain with solubles. ^d,e^ Means in the same column within the same item group with different superscripts are significantly different (*p* < 0.05).

**Table 3:** Least squares means and standard errors for the thickness of subcutaneous fat over longissimus, leg, and rump muscles.

Item	No. Lambs	Longissimus, mm	Leg, mm	Rump, mm
Protein, %				
12.5	12	6.1 ± 0.4	6.7 ± 0.5	18.5 ^c,d^ ± 1.8
15.7	12	5.0 ± 0.4	6.9 ± 0.5	22.3 ^c^ ± 1.9
18.9	12	5.3 ± 0.4	7.0 ± 0.6	16.6 ^d^ ± 1.9
Source				
SB ^a^	18	5.4 ± 0.3	7.7 ^c^ ± 0.3	19.2 ± 1.2
DD ^b^	18	5.3 ± 0.3	6.2 ^c^ ± 0.4	19.3 ± 1.3
Sex				
Wether	18	4.9 ^c^ ± 0.3	7.3 ± 0.4	19.9 ± 1.2
Ewe	18	5.9 ^d^ ± 0.3	6.6 ± 0.3	18.6 ± 1.2

^a^ SB, Soybean meal used as a protein source. ^b^ DD, Distillers dried grains with solubles used or partial replacement of SB. ^c,d^ Means in the same column within the same item group with different superscripts are significantly different (*p* < 0.05).

**Table 4:** Least squares means and standard errors for subcutaneous fat cell numbers and diameters of longissimus and rump mucle.

		Longissimus		Rump	
Item	Number Lambs	Number Cells ^a^	Diameter, mm ^b^	Number Cells ^a^	Diameter, mm ^b^
Protein, %					
12.5	12	195 ± 12	78.2 ± 2.0	188 ^f^ ± 12	79.9 ^e^ ± 2.3
15.7	12	231 ± 12	72.3 ± 2.0	231 ^e^ ± 12	72.3 ^f^ ± 2.3
18.9	12	220 ± 13	73.6 ± 2.1	211 ^e^ ± 13	77.1 ^e,f^ + 2.5
Sources					
SB ^c^	18	218 ± 9	74.6 ± 1.3	210 ± 8	75.0 ± 1.6
DD ^d^	18	225 ± 9	72.8 ± 1.3	212 ± 9	77.0 ± 1.7
Sex					
Wether	18	231 ± 9	71.7 ^e^ ± 1.3	224 ^e^ ± 8	74.3 ± 1.7
Ewe	18	212 ± 8	75.6 ^f^ ± 1.3	199 ^f^ ± 8	77.3 ± 1.6

^a^ No. of cells within 22.6 cm^2^ area. ^b^ Maximum round diameter. ^c^ SB = Soybean meal as protein source. ^d^ DD = Distillers dried grain with solubles used as a partial replacement for SB. ^e,f^ Means in the same column within the same item group with different superscripts are significantly different (*p* < 0.05).

## Data Availability

Data are contained within the article.
